# Structural Influences on Lithium-Ion Transport in Bismuth Oxides: A Molecular Dynamics Approach

**DOI:** 10.3390/ma18102287

**Published:** 2025-05-14

**Authors:** Seong-Beom You, Byeong Jun Kim, Yong Nam Ahn

**Affiliations:** School of Chemical, Biological, and Battery Engineering, Gachon University, Seongnam 13120, Gyeonggi, Republic of Korea

**Keywords:** Li-ion diffusion, bismuth oxide, solid electrolyte, molecular dynamics simulation, structural design

## Abstract

This study investigates Li-ion diffusion characteristics in Li-contained and Li-free bismuth oxide crystals, aiming to explore their potential as solid electrolytes for next-generation lithium-ion batteries. Although bismuth oxide has been widely applied as a solid electrolyte in fuel cells, its suitability for Li-ion battery applications remains unexplored. Using molecular dynamics simulations, we analyzed the Li-ion diffusion behavior in two distinct Li-contained bismuth oxide crystals with layered and non-layered structures, as well as four Li-free bismuth oxide phases. It is demonstrated that the layered structure exhibits a simpler and more organized diffusion pathway compared to the complex and bottlenecked pathways in the non-layered structure, resulting in superior Li-ion diffusivity. For Li-free bismuth oxide phases, diffusion coefficients vary significantly depending on structural characteristics, with the highest diffusion coefficient observed in the phase with minimal void fraction. A notable inverse relationship between void fraction and Li-ion diffusivity efficiency highlights the importance of structural design in enhancing ionic transport. This study provides valuable insights into the diffusion mechanisms of Li ions in bismuth oxide systems and offers strategic guidance for designing high-performance solid electrolytes, contributing to the advancement of all-solid-state battery technologies.

## 1. Introduction

Lithium-ion batteries (LIBs) are extensively used in diverse applications, including mobile electronics, electric vehicles (EVs), and grid-scale energy storage systems, due to their high energy density, long cycle life, and relatively low self-discharge rates [1,2,3,4,5,6,7,8]. Despite their widespread usage, LIBs have inherent limitations, including safety risks associated with flammable liquid electrolytes [9,10,11], limited energy density [12,13,14], and formation of lithium dendrite [15,16,17], which can lead to short-circuit failure. These issues have driven the exploration of next-generation battery technologies that can offer enhanced safety, higher energy density, and improved overall performance [18].

Among these emerging technologies, all-solid-state batteries (ASSBs) have attracted considerable attention. By replacing the liquid electrolyte with a solid-state counterpart, ASSBs offer numerous advantages, such as improved safety due to the elimination of flammable components, reduced dendrite formation, and the potential for greater energy density [19,20,21,22,23,24,25]. In particular, oxide-based solid electrolytes have gained significant research interest due to their high thermal stability, chemical resistance, and wide electrochemical stability windows [26,27,28,29]. Their resistance to dendritic lithium growth [30] and mechanical durability [31] also makes them particularly promising candidates for high-performance and long-lasting battery systems. However, oxide-based solid electrolytes suffer from relatively low ionic conductivity in comparison with sulfide-based or polymer-based solid electrolytes.

Various research efforts have focused on enhancing the ionic conductivity of oxide-based solid electrolytes [19,24,32,33,34,35]. For instance, a study on Ta-doped LLZO examined the effect of spark plasma sintering, showing notable improvements in ionic conductivity and densification [32]. In addition, Ouyang et al. recently studied the doping of LiFePO_4_ with chromium. In this study, the authors analyzed the effect of chromium doping on lithium-ion diffusion and demonstrated that the ionic conductivity increased by 24% compared to undoped samples. Similarly, Wang et al. recently explored various doping mechanisms in oxide and sulfide electrolytes. In this study, the authors analyzed the impact of different dopants on ionic conductivity and demonstrated significant improvements across the materials [34,35]. Despite recent advancements, further improvements in ionic conductivity are essential to enable the commercialization of oxide-based solid electrolytes for LIBs. This underscores the importance of exploring novel materials that have not yet been fully considered as potential solid electrolytes. In particular, gaining a deeper understanding of lithium-ion diffusion pathways within these materials is critical for guiding future research directions.

In this regard, our study investigates the lithium-ion diffusion characteristics of Li-contained bismuth oxide to explore its potential as a solid electrolyte for LIBs. Bismuth oxide has already been widely applied as a solid electrolyte in fuel cells and a lot of studies have demonstrated the excellent performance of bismuth oxide as a solid electrolyte [36,37,38,39,40]. For example, Ahn et al. [37] developed a bilayered electrolyte composed of ceria and bismuth oxide, which enabled high power output even at reduced operating temperatures in low-temperature solid oxide fuel cells. In a similar approach, Oh et al. [40] designed a functionally graded bilayer structure integrating Bi_2_O_3_ and ZrO_2_. This configuration simultaneously ensured high ionic conductivity and mechanical stability, thereby improving both electrochemical performance and long-term durability in solid oxide fuel cells. Although bismuth oxide has been widely applied in fuel cells due to its high performance as a solid electrolyte, to the best of the authors’ knowledge, its application as a solid electrolyte for lithium-ion batteries (LIBs) has not yet been explored. Conventional approaches to enhancing the performance of solid electrolytes for LIBs, such as simple compositional tuning via doping, have shown limited success. Therefore, incorporating lithium into bismuth oxide structures presents a promising opportunity to achieve high performance in lithium-ion battery applications. Using molecular dynamics simulations, we analyze how the crystal structure of Li-contained bismuth oxide affects lithium-ion diffusion coefficients and pathways. Furthermore, we propose specific crystal structures capable of enhancing lithium-ion diffusivity, offering valuable insights for the design of next-generation LIBs.

## 2. Simulation Details

**Figure 1:**
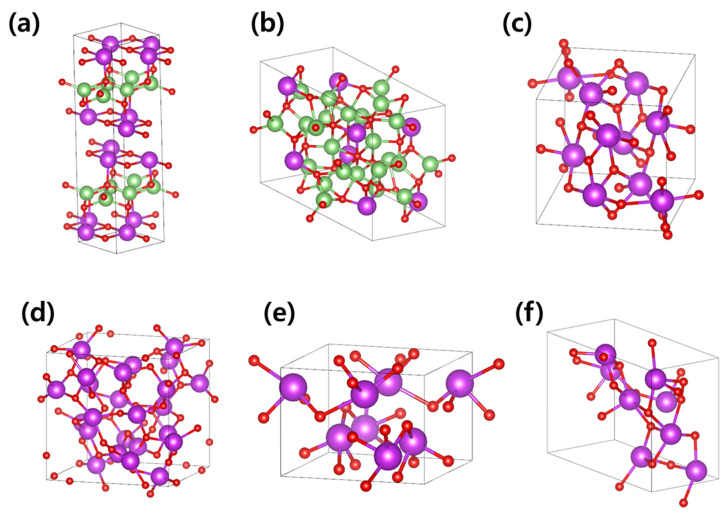
Atomic structures of (**a**) LiBiO_2_, (**b**) Li_3_BiO_3_, and Bi_2_O_3_ with (**c**) monoclinic, (**d**) cubic, (**e**) tetragonal, and (**f**) triclinic structures. Li, Bi, and O atoms are represented in green, purple, and red, respectively.

We examined six experimentally observed crystal structures of Li-contained bismuth oxide obtained from the Materials Project database [41]. These six structures can be broadly categorized into two groups based on the arrangement of lithium sites: layered structures, where lithium ions are arranged in a stacked configuration, and non-layered structures, where lithium ions exhibit no specific directional arrangement. For our analysis, one representative structure from each category was selected to investigate lithium-ion diffusion. The layered structure (Figure 1a) corresponds to the chemical formula LiBiO_2_ with a space group of Ibam (#72, Orthorhombic), while the non-layered structure (Figure 1b) has the chemical formula Li_3_BiO_3_ with a space group of P-1 (#2, Triclinic).

Additionally, during the synthesis of Li-contained bismuth oxide, lithium-free bismuth oxide phases inevitably form and coexist as polycrystalline structures within the solid electrolyte. To account for this, we selected four lithium-free bismuth oxide structures with monoclinic, cubic, tetragonal, and triclinic structures (Figure 1c–f). By introducing lithium ions into the interstitial sites of these structures, we investigated how lithium-diffusion characteristics vary depending on the crystal structure.

It is worth noting that it is essential to investigate lithium-ion diffusion not only within perfect crystal structures but also along grain boundaries for a comprehensive understanding of ionic conductivity in polycrystalline systems. However, the present study focuses on elucidating the fundamental diffusion mechanisms within ideal crystal structures. This serves as a necessary first step for systematically exploring the more complex diffusion behavior at grain boundaries. In particular, grain boundaries in polycrystalline materials can exhibit a wide variety of atomic arrangements, and their influence on lithium diffusion is strongly dependent on the specific grain boundary structure. Therefore, to accurately assess diffusion phenomena occurring at grain boundaries in bismuth oxide systems, a detailed investigation of the types of grain boundaries that can realistically form in these systems is required. Such an investigation falls beyond the scope of the current study and is currently being pursued as a follow-up to this work.

In Li-free bismuth oxide, lithium ions can occupy only a limited number of interstitial sites, resulting in a significantly lower concentration of Li ions compared to that in Li-contained bismuth oxide. When analyzing the dynamic properties of Li ions via molecular dynamics (MD) simulations, such a low ion concentration poses a challenge in achieving sufficient statistical reliability. Therefore, a large number of MD simulations is required to ensure robust statistical sampling. Since ab initio MD simulations impose a substantial computational cost, making them impractical for extensive sampling, the diffusion characteristics of Li ions, such as the diffusion coefficient and diffusion pathway within the Li-free bismuth oxides, are analyzed using classical MD simulations. The interatomic potentials are based on the force field proposed by Pedone et al. [42], which has been extensively validated and widely applied in the analysis of mechanical properties and diffusion behavior across various oxide systems. The potential parameters for the Li-O and O-O pairs are obtained from ref. [43,44]. For the Bi-O pair, applying different potential parameters for each crystal structure is impractical. Instead, the Bi-O potential parameters are optimized to reproduce the structural and mechanical properties of amorphous Bi_2_O_3_ as predicted by ab initio MD simulations. Both the ab initio and classical MD simulations followed the same melt–quench procedure [45] to generate amorphous structures. In the ab initio MD, the system consisted of 80 atoms in a simulation cell of dimensions 12 × 17 × 7 Å^3^. To evaluate the transferability and robustness of the optimized potential, classical MD simulations were performed using a significantly larger model constructed by replicating the original cell 5 × 5 × 5 times, resulting in a system with 10,000 atoms. The estimated properties of the amorphous Bi_2_O_3_ by using the optimized potential parameters are almost identical with those from the ab initio MD simulations (see Table 1). The potential parameters utilized in this study are summarized in Table 2.

To analyze the diffusion characteristics of Li ions in Li-contained bismuth oxide, ab initio MD simulations are performed by using Vienna ab initio Simulation Package (VASP v5.4.4) [46]. The Perdew–Burke–Ernzerhof (PBE) function of the generalized gradient approximation (GGA) is adopted for the exchange and correlation contributions [47]. Core electrons are incorporated into the pseudopotentials using the projector augmented wave (PAW) method [48]. A plane-wave cutoff energy of 520 eV is used with a 2 × 2 × 2 k-mesh in terms of the Monhorst-Pack scheme [49] for Brillouin zone integration. The energy convergence criterion for the relaxation of the electronic degrees of freedom is set to ≤10^−5^ eV, and time step is 1 fs. A 500 ps production run is conducted to analyze the dynamic properties of Li ions in each system. Prior to the production run, the system is equilibrated for 100 ps. During the equilibration stage, the total energy of the system converges to a stable value as shown in Figure S1a, confirming that the system reaches equilibrium before the production run.

The dynamic properties of Li ions in Li-free bismuth oxide are explored by using classical MD simulations. A 100 ps equilibration is also performed prior to the 500 ps production run, and the total energy of the system converges stably during the equilibrium stage (see Figure S1b). To maintain the integrity of the crystal structures during classical MD simulations at high temperature conditions, spring forces ($F_{s}$) are applied to Bi and O atoms in the simulation cell, as

(1) $$F_{s}=k_{s}∆r,$$

where $k_{s}$ is spring constant and ∆r is the coordination change of an atom from its initial position. Since the atomic positions do not change significantly from their initial states within a 1 fs timestep, both the displacement (∆r) and the resulting force ($F_{s}$) remain small. Moreover, since the spring force is not applied to Li ions, the forces acting on Bi and O atoms have only a limited influence on the dynamics of Li ions. In addition, an identical spring constant ($k_{s}=20\;eV/{Å}^{2}$) is used for all crystal structures, ensuring that the relative comparison of Li-ion diffusion characteristics between different structures remained unaffected by the spring force. It should be noted that the harmonic spring force stabilizes the crystal framework beyond the melting temperature. This non-physical constraint is intentionally introduced to prevent the solid from melting at high temperatures, which allows for the acceleration of ion dynamics without phase transformation. The aim of this setup is to enable a qualitative comparison of Li-ion diffusion behavior across different Bi_2_O_3_ structures, rather than to reproduce experimentally accurate diffusion coefficients.

All classical MD simulations are performed by using large-scale atomic/molecular massively parallel simulator (LAMMPS 2Aug2023 version) package [50] with 1 fs integration time step. During the simulation, the pressure of the system is kept constant at 1 atm with Parrinello–Rahman barostat [51] and the temperature is controlled by utilizing a Nosé–Hoover thermostat [52].

It is worth mentioning that performing MD simulations at room temperature facilitates direct comparison with experimental data and provides more practical insight into the development of solid electrolytes for LIBs. However, the inherent timescale of typical MD simulations is generally restricted to tens of nanoseconds or less. At such timescales, atomic diffusion in solid phases at room temperature is often too slow to be meaningfully captured. In contrast, simulations conducted at elevated temperatures (above 500 K) enhance atomic mobility by providing sufficient thermal energy, and result in statistically reliable diffusion data within a few hundred picoseconds. Since the primary focus of this study is not on the quantitative prediction of dynamic properties but rather on the qualitative comparison of Li-ion diffusion behavior as a function of Bi_2_O_3_ structure, all MD simulations in this study are performed at elevated temperatures (above 500 K) to strengthen the reliability of relative comparisons between different Bi_2_O_3_ structures.

## 3. Results and Discussion

### 3.1. Li-Contained Bismuth Oxide

To analyze ion migration pathways in Li-contained bismuth oxide crystals, bond valence maps are utilized [53,54]. The bond valence between a cation (Li) and an anion (O), $S_{M-X}$, is computed using Equation (2),

(2) $$S_{M-X}=exp\left[\frac{R_{0}-R}{b}\right]$$

where R is the actual bond length [55]. The empirical parameters $R_{0}$ and b for the Li–O pair are 1.1725 and 0.515, respectively [56]. By summing the bond valences of all Li–O pairs within a specified cut-off distance of 8 Å at each point on a three-dimensional grid with a mesh size of 0.1 Å, a bond valence map can be constructed for Li ions. Locations on the map where the summed bond valence approximates the ideal valence of Li ions (+1) are considered the most likely sites for Li ions to reside. The deviation of the bond valence sum from the ideal valence is defined as $∆V_{B}=\left|V_{M}-V_{i}\right|$. Here, $V_{M}$ is the bond valence sum and $V_{i}$ corresponds to the ideal valence of Li ions (i.e., $V_{i}=+1$). Li-ion transport predominantly occurs along pathways where $∆V_{B}$ remains minimal. These migration pathways can be identified as regions on the grid where $∆V_{B}$ is small.

**Figure 2:**
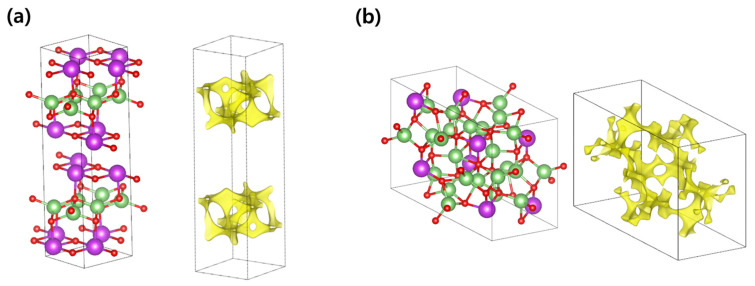
Atomic structures and corresponding bond valence isosurface for (**a**) LiBiO_2_ and (**b**) Li_3_BiO_3_. Here, the bond valence isosurfaces are presented for $∆V_{B}=0.1$.

Figure 2 shows the bond valence isosurface for $∆V_{B}=0.1.$ The layered structure (hereafter referred to as LS) presents a simple and straightforward diffusion path, primarily localized within the Li layer (see Figure 2a). When periodic boundary conditions are considered, it suggests that Li ions predominantly undergo active two-dimensional diffusion within this layer. In contrast, the nonlayered structure (hereafter referred to as NLS) demonstrates a three-dimensional diffusion network within the crystal structure (see Figure 2b). When the network features well-connected and low-resistance pathways, such a three-dimensional structure can facilitate multi-directional Li diffusion, potentially resulting in a higher ionic diffusion coefficient compared to two-dimensional systems. However, the network observed in NLS exhibits a highly complex structure, characterized by pronounced curvatures and numerous bottlenecks along the pathways. This intricate diffusion path limits the mobility of Li ions, making it challenging to achieve a high diffusion coefficient despite the formation of a three-dimensional network.

Bond valence analysis is a useful method for examining ion diffusion characteristics due to its low computational cost. However, since it is based on the analysis of static structures, it does not account for kinetic factors. Since ion diffusion is a highly dynamic physical and chemical phenomenon, ignoring the influence of kinetic factors can lead to incomplete or inaccurate interpretations. To address this limitation, molecular dynamics (MD) simulations were employed to calculate the Li-ion diffusion coefficients in both LS and NLS.

Diffusion coefficients can be calculated from mean squared displacement (MSD) of Li ions, defined as

(3) $$MSD=\langle {\left(x(t)-x_{0}\right)}^{2}\rangle$$

where x(t) is the position of a Li ion at time t and $x_{0}$ is the initial coordinate of the Li ion. Then, the diffusion coefficient (D) is calculated from the slope of the time–MSD curves as

(4) $$D=\frac{MSD}{6t}\;$$

**Figure 3:**
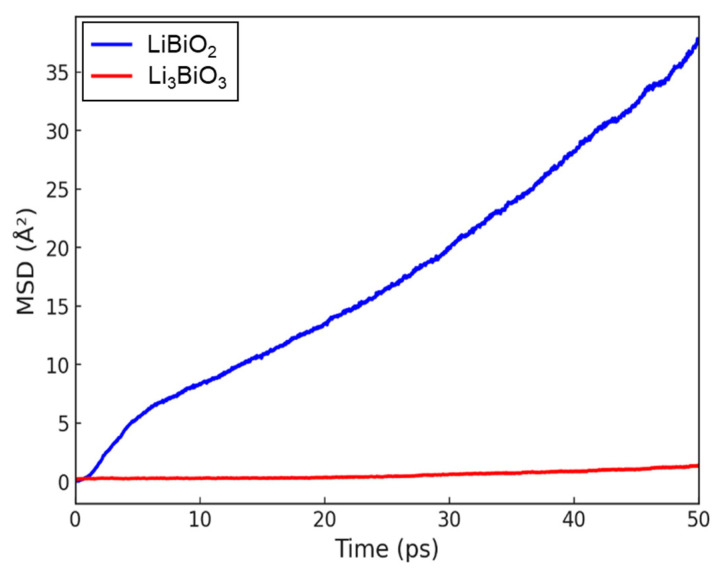
Mean squared displacement (MSD) of Li ions within LiBiO_2_ and Li_3_BiO_3_ structures at 1000 K.

Figure 3 shows the obtained time–MSD curves of Li ions for LS and NLS at 1000 K. These curves are obtained at three different temperatures (500, 1000, and 1500 K), and the corresponding diffusion coefficients are summarized in Table 3. At all temperatures, LS exhibits a higher Li-ion diffusion coefficient compared to NLS. The difference in diffusion coefficients between the two structures is most pronounced at lower temperatures and gradually diminishes as the temperature increases. The lower diffusion coefficient in NLS suggests that the activation energy required for Li-ion hopping is higher than in LS. At higher temperatures, sufficient thermal energy is available to overcome the activation barriers in NLS, reducing the impact of its high activation energy on ion mobility.

The observation that LS maintains a higher diffusion coefficient at all temperature conditions aligns well with the bond valence analysis results. This indicates that the Li-ion diffusion pathways observed in bond valence analysis (Figure 2) likely correspond well to the actual diffusion path. To explore the diffusion pathways of Li ions during MD simulations, the simulation cell is divided into a cubic grid with 50 divisions along each axis, resulting in a total of 12,500 grid cells. At each time step, the positions of all Li ions are mapped onto the corresponding grid points. This mapping process is repeated over a 10 ps simulation. Then, the number of Li ions mapped to each grid point is counted to calculate the probability distribution of Li-ion presence at each point. The diffusion pathway of Li ions can be identified by the grid points with high probability.

**Figure 4:**
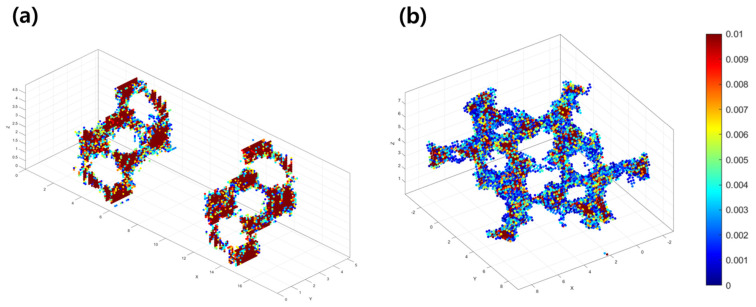
Spatial probability density of Li ions within (**a**) LiBiO_2_ and (**b**) Li_3_BiO_3_ during 10 ps of MD simulations. The color scale represents the normalized probability density, i.e., red regions correspond to high-probability locations, while blue regions indicate low-probability areas.

Figure 4 presents the obtained probability distribution of Li ions in LS (Figure 4a) and NLS (Figure 4b) during MD simulations for 10 ps. The overall profile of the probability distribution closely aligns with the bond valence isosurface, indicating that the structural factors of the crystal framework play a dominant role. This means that kinetic factors, such as diffusion rates, exert a limited influence and are insufficient to significantly alter the diffusion pathways. The obtained probability distributions reveal distinct characteristics between LS and NLS. In the case of LS, diffusion pathways are dispersed throughout the entire simulation cell, exhibiting a tortuous and irregular pattern. In contrast, NLS shows diffusion pathways confined strictly to the Li-ion layers, with a consistent and regular channel width. The simpler and more organized diffusion path observed in NLS facilitates more efficient Li-ion transport compared to LS. In fact, the diffusion path in NLS (Figure 4b) has the higher density of red-colored grid points than LS (Figure 4a), indicating greater probabilities of Li-ion presence along the pathways.

### 3.2. Li-Free Bismuth Oxide

Although Li-containing bismuth oxides constitute a significant portion of solid electrolytes, the synthesis process inevitably leads to the formation of Li-free bismuth oxide phases, whose structural characteristics such as atomic arrangements and bonding environments can significantly influence Li-ion diffusion pathways and efficiency. Therefore, understanding how these factors affect Li-ion diffusivity will provide essential insights for minimizing diffusion-inhibiting structures and guiding the design of high-performance solid electrolytes. In this regard, we considered four different Li-free bismuth oxide crystals, as shown in Figure 1c–f. These crystals exhibit distinct crystal structures: monoclinic, cubic, tetragonal, and triclinic, which we will refer to as NL1, NL2, NL3, and NL4, respectively. The formation energies of Li interstitial defects in these four different crystal structures. As shown in Table S1, all four structures exhibit negative formation energies, indicating that Li ions can be accommodated in interstitial sites within these bismuth oxide phases. To analyze the diffusion of Li ions within each crystal structure, Li ions are introduced into the interstitial sites of the crystals, and their diffusion coefficients are estimated from MD simulations at 1000 K.

**Figure 5:**
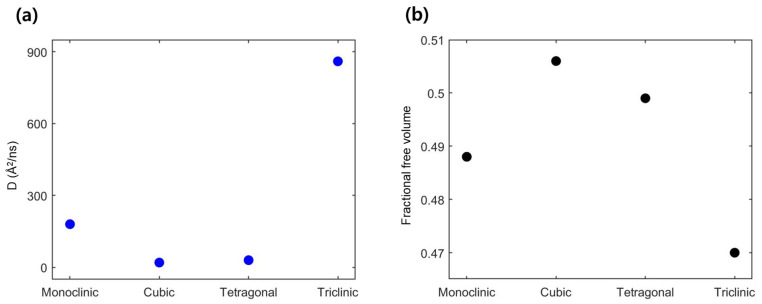
(**a**) Diffusion coefficients of Li ions and (**b**) fractional free volume within monoclinic, cubic, tetragonal, and triclinic Bi_2_O_3_ crystal structures.

Figure 5a shows that the calculated diffusion coefficients decrease in the order of NL4 > NL1 > NL3 > NL2. Notably, NL4 exhibits a significantly higher diffusion coefficient compared to the other crystals, while NL2 and NL3 show values that are approximately two orders of magnitude lower than NL4. Interestingly, these diffusion coefficients exhibit an inverse relationship with the void fraction within each crystal structure (see Figure 5b). Here, the void fraction is defined as

(5) $$void\;fraction=\frac{1}{V_{cell}}\left[V_{cell}-\frac{4}{3}\pi \left(N_{Bi}r_{Bi}^{3}+N_{O}r_{O}^{3}\right)\right]$$

where $V_{cell}$ is the volume of the simulation cell, $N_{Bi}$ and $r_{Bi}$ are the number and ionic radius of bismuth ions, respectively, and $N_{O}$ and $r_{O}$ are the number and ionic radius of oxygen ions, respectively. In Li-free bismuth oxide, there are no stable Li sites within the crystal structure. As a result, ions tend to rapidly escape from unstable interstitial sites, leading to a higher diffusion coefficient. When the void fraction is large, sufficient space around these interstitial sites becomes available, which reduces the driving force for ion escape. The Li interstitial formation energies presented in Table S1 also reflect this trend, where structures with larger void fractions exhibit lower formation energies. This suggests that a larger void fraction leads to a more stabilized interstitial site for Li, resulting in a higher energy barrier for Li to escape from the site. Consequently, a higher void fraction leads to a lower diffusion coefficient.

This relationship between the void fraction and the diffusion coefficient provides valuable insights for designing high-ion-conductivity bismuth oxide-based solid electrolytes. For instance, amorphous solids generally have lower density and higher void fractions compared to crystalline structures, which negatively affect ion diffusion. Therefore, optimizing process parameters, such as the melt–quench rate, to suppress the formation of the amorphous phase could enhance the ionic conductivity of solid electrolytes.

## 4. Conclusions

This study provides critical insights into the diffusion behavior of Li ions in Li-contained and Li-free bismuth oxide crystals. Through MD simulations, we systematically explored how structural characteristics influence Li-ion transport pathways and diffusion coefficients. In Li-contained bismuth oxide crystals, the analysis revealed that layered structures (LSs) exhibited a simpler and more organized diffusion path compared to the complex and bottlenecked pathways in non-layered structures (NLSs). The bond valence analysis and MD simulations consistently showed that LS supports more efficient lithium-ion transport, resulting in superior Li-ion diffusivity. For Li-free bismuth oxide crystals, diffusion coefficients varied significantly across the analyzed structures, decreasing in the order of NL4, NL1, NL3, and NL2. The remarkably high diffusion coefficient of NL4 and the substantially lower values observed in NL2 and NL3 (approximately two orders of magnitude lower) underscore the structural complexity of lithium-ion migration pathways in these phases. The inverse relationship between void fraction and diffusion coefficient indicates the reduced tendency for Li ions to escape unstable interstitial sites in structures with larger void fractions.

While this study focuses on a qualitative comparison of Li-ion diffusion behavior across different crystal structures, it sets the stage for more quantitative investigations. Future studies may involve the development of advanced interatomic potentials capable of capturing grain boundary effects, phase transitions, and temperature-dependent properties in polycrystalline systems. Such comprehensive approaches will be essential for evaluating the potential of bismuth oxide as a solid electrolyte for LIBs. Nonetheless, as an initial exploration into a novel material system, we believe that this study provides a valuable foundation for future research.

## Figures and Tables

**Table 1:** Structural and mechanical properties of amorphous Bi_2_O_3_ estimated by ab initio and classical MD simulations.

Method	Bond Length (Å)			Density (g/cm^3^)	Young’s Modulus (GPa)	Poisson Ratio
	Bi–Bi	Bi–O	O–O			
Ab initio MD	3.6	2.2	3.0	9.1	71.0	0.26
Classical MD	3.5	2.2	3.1	10.2	67.5	0.28

**Table 2:** Potential parameters for Li–O, Bi–O, and O–O pairs. Exponents on Li, Bi, and O refer to their charge values for Coulombic interactions.

	*D_ji_* (eV)	*a_ij_* (Å^−2^)	*r*_0_ (Å)	*C_ij_* (eV Å^12^)
Li^0.6^–O^−1.2^	0.001114	3.429506	2.681360	1.0
Bi^1.8^–O^−1.2^	0.000132	2.01300	4.301589	3.0
O^−1.2^–O^−1.2^	0.042395	1.379316	3.618701	22.0

**Table 3:** Diffusion coefficients of Li ions within LiBiO_2_ and Li_3_BiO_3_ structures at 500 K, 1000 K, and 1500 K.

Temperature (K)	Diffusion Coefficient (Å/ns)	
	LiBiO_2_	Li_3_BiO_3_
500	71.3	1.7
1000	171.3	15.5
1500	320.5	64.0

## Data Availability

The original contributions presented in this study are included in the article/Supplementary Material. Further inquiries can be directed to the corresponding author.

## Supplementary Materials

The following supporting information can be downloaded at: https://www.mdpi.com/article/10.3390/ma18102287/s1. Figure S1. Total energy as a function of time during the equilibration process for (a) LiBiO_2_ system (AIMD) and (b) monoclinic Bi_2_O_3_ system (classical MD); Table S1. Formation energies of a Li interstitial defect for four Li-free bismuth oxide structures.
