# Supplementary material for: Structural Influences on Lithium-Ion Transport in Bismuth Oxides: A Molecular Dynamics Approach
Source: Materials (Basel). 2025 May 14;18(10):2287. doi: 10.3390/ma18102287 (PMC12113621; doi:10.3390/ma18102287)
Supplement: Supplementary file 1 [file materials-18-02287-s001.zip › materials-3584548-supplementary.pdf]

Supporting Information

Communication

Structural Influences on Lithium-Ion Transport in Bismuth Oxides: A Molecular Dynamics Approach

Seong-Beom You, Byeong Jun Kim and Yong Nam Ahn *

School of Chemical, Biological, and Battery Engineering, Gachon University, Seongnam 13120, Gyeonggi, Republic of Korea
* Correspondence: yahn@gachon.ac.kr

Equilibration process

Before the production runs using ab initio molecular dynamics (AIMD) and classical molecular dynamics (classical MD) simulations, all systems considered in this study are equilibrated for 100 ps. Fig. S1a and Fig. S1b show the total energy as a function of time during the equilibration process for the $LiBiO_2$ system (AIMD) and the monoclinic Bi_2O_3 system (classical MD), respectively. As shown in the figures, the total energy converges to a stable value within 100 ps, indicating that the systems reach equilibrium during the equilibration process. Similar energy convergence behavior is observed for all systems considered in this study.

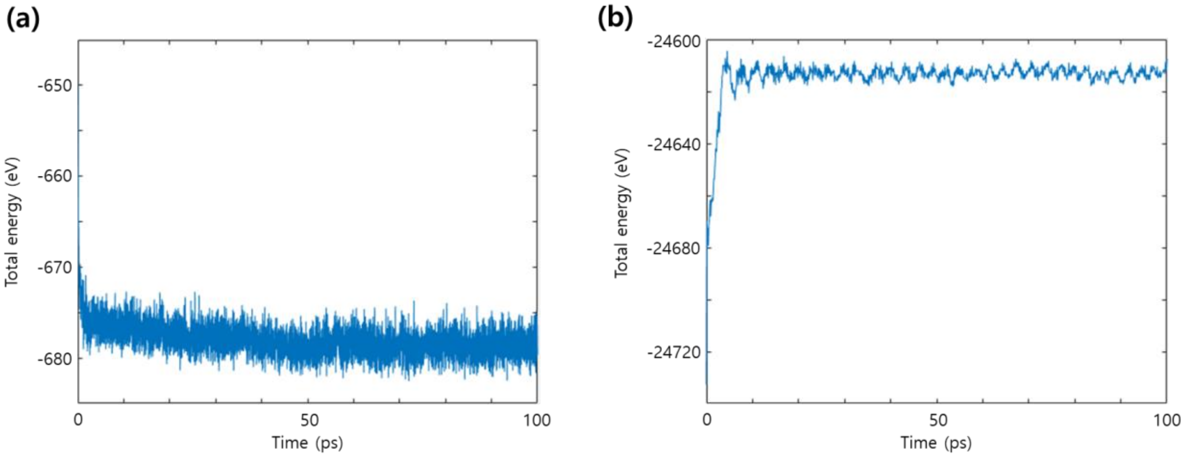

Figure S1. Total energy as a function of time during the equilibration process for (a) $LiBiO_2$

system (AIMD) and (b) monoclinic Bi_2O_3 system (classical MD)

Formation energy of Li interstitial defects in Li-free bismuth oxides

The formation energy of a Li interstitial defect in Li-free bismuth oxide structures is calculated by

$$E_{\mathrm{F}} = E_{\mathrm{total}} - E_{\mathrm{Bi2O3}} - \mu_{\mathrm{Li}}$$

where E_{F} is the formation energy, E_{total} is the total energy of the $2 \times 2 \times 2$ supercell containing one Li interstitial, E_{Bi2O3} is the total energy of the pristine bismuth oxide supercell, and μ_{Li} is the chemical potential of Li. Table S1 shows the calculated formation energy for four different Li-free bismuth oxide crystal structures.

Table S1. Formation energies of a Li interstitial defect for four Li-free bismuth oxide structures

**Structures**	NL1 (Monoclinic)	NL2 (Cubic)	NL3 (Tetragonal)	NL4 (Triclinic)
**Formation energy (eV)**	-0.99	-1.65	-1.94	-0.54
